# Case of Gun-Shot Wound of the Knee-Joint; Ball Lodged in the Head of the Tibia; Extracted through the Joint Six Months after the Accident; Recovery with a Useful Limb

**Published:** 1864-07

**Authors:** R. A. Kinloch

**Affiliations:** Surgeon P. A. C. S., Charleston, S. C.


ART. III.—*Case of Gun-Shot Wound of the Knee-Joint.—Ball lodged in the Head of the Tibia.—Extracted through the Joint Six Months after the Accident.—Recovery with a Useful Limb.* By R. A. KINLOCH, Surgeon P. A. C. S., Charleston, S. C.

Private L. C., aged 17 years, Sixth South Carolina regiment, was wounded at the battle of the Seven Pines on the 31st of May, 1862. On the 23d of November, while on a visit to Chester District, S. C., I was requested to see him in consultation with his attending surgeon. I received the following history of his case: He was wounded at the time above specified, while lying on his back, re-loading his gun, with his knees slightly flexed. Was examined by a surgeon on the field, who remarked that his leg would have to be amputated, but who gave no comprehensive opinion as to the direction and extent of the wound, or the position of the ball. Patient walked some distance after he was shot without experiencing very great pain. The following day he was sent to the St. Charles hospital, at Richmond. The limb was then much tumefied and very painful. The ball had entered upon the inner side of the right knee, apparently, a line or two below the articulation. There was no orifice of exit. In the opinion of most of the examining surgeons the ball had lodged; but a few of them, after the progress of the case, were inclined to believe that it had escaped through the orifice of entrance. Consultation developed further differences of opinion as to the precise nature of the wound, the implication of the joint, and the position of the foreign body. It was, however, determined not to interfere by any operative procedure. (The details of treatment need not here be given.) The patient remained in hospital until the 3d of August, never leaving his bed, and scarcely ever changing from the supine position; he had suffered continually from fever, and during a portion of time from traumatic delirium. Finally, he was moved upon a hand-litter to the railroad station, and then to home in Chester. He continued bed-ridden, without experiencing any acute suffering, after recovering from the effect of his journey, until the middle of October. At this time he left his bed and tried the use of crutches. After but little exercise he was compelled to take to bed again, and was a week in recovering from a very painful and inflamed condition of the limb, engendered by the efforts he had made. This was repeated a second and third time with a like result, and, finally, erysipelas attacked the parts about the knee, and extended high up the thigh. He had been, during most of this time, abandoned to expectation with good nourishing diet, and had gained flesh and strength while enjoying the comforts of home; the irritation of the knee, however, had always persisted in a sub-acute form, and the wound had continued to discharge. The amount of discharge varied at different times; the suffering being always less when the discharge was free.

At the time of my visit, (23d November,) patient's general health was good; he was, however, despondent; feared that he would never have the use of the limb, and was willing to have it removed, if I thought it advisable, to rid him of his suffering. The limb was flaccid and attenuated. A fistulous opening upon the inner side of the joint, just below the articular margin of the tibia, indicated the original orifice of entrance; this was discharging a thin pus. The joint was imperfectly anchylosed; the leg very slightly flexed upon the thigh; the tissues about the knee were thickened, and the integument of the thigh and part of that of the leg, indicated a recent attack of erysipelas that had been treated by the local application of the nitrate of silver.

A careful consideration of all the circumstances of the case led me to the following views and practice: As the projectile had come from behind and from the left of the patient, while he was upon his back with the legs in easy flexion, it had probably ranged downwards and outwards. There was no trace of it discoverable in the soft parts. The open wound, continuous discharge, and perpetual irritation, induced me to believe that the ball was imbedded in the tibia. The knee-joint was disorganized and anchylosed from traumatic inflammation. But had the ball lodged in the cavity of the joint? or had it even perforated the capsule in reaching the head of the tibia? The first question I disposed of readily, by remembering that the patient had walked with ease after the reception of the injury, and had not afterwards suffered the excruciating symptoms that usually attend the presence of a foreign body within the joint. The second question was harder of solution. The traumatic inflammation of the joint could be accounted for as readily upon the supposition of the ball having penetrated the tibia and fractured it through to the joint, as by its supposed passage through the capsule. Fortunately, the correct interpretation of the disorganized state of the joint was not of great practical importance. It was enough to know that the disorganization existed. This knowledge inculcated the belief that the foreign body might be sought *by way of the joint,* without seriously jeopardizing the patient. I conceived that the cutting into this joint would not develop the symptoms that are expected to ensue from cutting into a joint whose tissues approach the normal condition. I determined then to search for the foreign body, and to be prepared, if necessary, even to resect the joint. My diagnosis was only inferential, but I considered more positive knowledge only valuable inasmuch as it might enable me more promptly and certainly to get upon the track of the ball.

On the 29th of November, the patient being fully chloroformed, I performed the following operation: 1st.—A semilunar flap of integuments, which include the fistulous opening, was dissected and turned up from the deeper tissues over the inner side of the joint. 2d.—I proceeded with the finger, and afterwards with a large probe, to ascertain the direction of the fistulous track through the deep tissues. I soon discovered that the track could be traced through the lateral ligament of the joint to the depth of several inches. 3d.—I incised the lateral ligament freely, and passing my finger through the opening, I discovered and turned out a piece of necrosed bone about the size of the last phalanx of the finger. This was a portion of the articulating surface of the tibia, which had been detached by the passage of the ball. After its removal, my finger occupied a notch or groove upon the articular surface of the tibia, and could be forced on downwards and outwards towards the head of the fibula. As the joint, however, was but slightly flexed, the finger could only advance to a certain depth, because of the inner condyle of the femur. Feeling that I was certainly upon the track of the foreign body, I seized the leg of the patient with my left hand, and forcibly flexed the joint until I found that my index finger, which had not been removed from the wound, could by an effort be made to pass on under the articulating surface of the inner condyle of the femur. Upon instituting the flexion some of the bony adhesions snapped asunder with a noise, and my finger was pushed on into a deep cavity in the outer portion of the head of the tibia, and rested upon the foreign body. 4th.—The finger was withdrawn, and a pair of bullet forceps introduced along the track. At the first effort the ball was seized and extracted with great ease. It proved to be a large minie, much flattened, and having a piece of the patient's pantaloons attached. A sponge full of warm water was thrown two or three times into the joint, to wash out the debris from the cavity in which the ball had rested. The joint was found to be completely disorganized. The synovial membrane, interatricular cartilages, and crucial ligaments could scarcely be recognized, and the articular surfaces of the bones were deeply eroded.

At this stage of the operation it became a question whether it might not be better practice to get rid of the diseased surfaces, by resecting the joint. I felt apprehensive less persistence of chronic inflammation in those tissues of low vitality might prevent recovery with a useful limb. I entertained serious misgivings as to the ultimate establishment of complete bony anchylosis, which I thought essential for the well-being of the patient. Having met with uniform success in the operation of resection of the knee for chronic disease, I favored this operation, and would have practised it could I have had the after management of the case. But the necessity I was under for leaving the locality the same day, together with the adverse opinions of my medical friends, decided me to interfere no farther. The tegumentary flap was brought into position and secured by three points of suture, only the dependent corner of the wound being kept open by a tent; the limb was extended upon a bolster; cold water dressing was applied; and a full anodyne administered. I learned that the case progresed quite satisfactorily. But little general or local excitement followed the operation. The wound healed kindly, and in three weeks the patient experienced *"a feeling of relief that he had not known since the accident."* In a letter received from the patient, dated April 23d, 1864, giving me account of his progress since the operation, he says:

"The only drawback to my convalescence was an attack of erysipelas, in Jan, 1863, which I attributed to imprudence in eating. On the 17th of February, 1863, I began to move about on crutches, the wound having entirely healed before this. Since then, the limb has gained rapidly in strength. In May I laid aside my crutches and took to using a stick. I can now walk a mile without resting, and often walk some without my stick. My joint is stiff, but I think I shall some day be able to run. I can now run a few steps, and can truly say the leg is well. I practise walking without my stick as much as I can."

